# Frequency, factors and costs associated with injection site infections: Findings from a national multi-site survey of injecting drug users in England

**DOI:** 10.1186/1471-2334-8-120

**Published:** 2008-09-18

**Authors:** Vivian Hope, Jo Kimber, Peter Vickerman, Matthew Hickman, Fortune Ncube

**Affiliations:** 1 Centre for Infections, Health Protections Agency, London, UK; 2 Centre for Research on Drugs & Health Behaviour, London School of Hygiene & Tropical Medicine, London, UK; 3 Department of Social Medicine, University of Bristol, Bristol, UK; 4 National Centre in HIV Epidemiology and Clinical Research, University of New South Wales, Sydney, Australia

## Abstract

**Background:**

Injection site infections among injecting drug users (IDUs) have been associated with serious morbidity and health service costs in North America. This study explores the frequency, factors and costs associated with injection site infections among IDUs in England.

**Methods:**

Unlinked-anonymous survey during 2003/05 recruiting IDUs from community settings at seven locations across England. Self-reported injecting practice, symptoms of injection site infections (abscess or open wound) and health service utilisation data were collected using a questionnaire, participants also provided dried blood spot samples (tested for markers blood borne virus infections). Cost estimates were obtained by combining questionnaire data with information from national databases and the scientific literature.

**Results:**

36% of the 1,058 participants reported an injection site infection in the last year. Those reporting an injection site infection were more likely to be female and aged over 24, and to have: injected into legs, groin, and hands in last year; injected on 14 or more days during the last four weeks; cleaned needles/syringes for reuse; injected crack-cocaine; antibodies to hepatitis C; and previously received prescribed substitute drug. Two-thirds of those with an injection site infection reported seeking medical advice; half attended an emergency department and three-quarters of these reported hospital admission. Simple conservative estimates of associated healthcare costs range from £15.5 million per year to as high as £30 million; though if less conservative unit costs assumptions are made the total may be much higher (£47 million). The vast majority of these costs are due to hospital admissions and the uncertainty is due to little data on length of hospital stays.

**Conclusion:**

Symptoms of injection site infections are common among IDUs in England. The potential costs to the health service are substantial, but these costs need more accurate determination. Better-targeted interventions to support safer injection need to be developed and evaluated. The validity of self-reported symptoms, and the relationship between symptoms, infection severity, and health seeking behaviour require further research.

## Background

A range of bacteria can infect injecting drug users' (IDUs) injection sites, resulting in illnesses ranging from localised skin and soft tissue infections to systemic and toxin producing infections [1,2]. These infections can result in serious morbidity requiring inpatient intervention (e.g. intensive intravenous antibiotics, surgical debridement, amputation) and sometimes death [1,2]. The reported prevalence of recent or current infections such as abscesses, infected ulcers and cellulitus among IDUs ranges from under one in 10 in Australia [3] to between one in five and one in three in North America and Europe [4-6]. These infections are also the most common presenting diagnoses among IDUs attending emergency departments in North America [7,8]. Injection site infections are associated with poor hygiene and unsafe injection practices including inadequate cleaning of the hands or the injection site, needle and syringe re-use, multiple injection attempts, use of multiple injection sites, subcutaneous injection, and drawing blood back into the syringe repeatedly [3,5,9,10].

There has been increasing concern in the United Kingdom (UK) about the extent of bacterial infections among IDUs [11-13]. Reports of severe group A streptococci infections among IDUs in the UK have increased over ten fold in recent years from less than ten per annum in the mid 1990's to 143 per annum in 2004 [11]. There have also been reports of community acquired Meticillin resistant *Staphylococcus aureus *infection among IDUs in recent years [14,15], and outbreaks of tetanus and wound botulism [16,17].

Little is known about the extent of injection site infections, or the factors associated with them, among IDUs in England. These infections are, however, likely to place a considerable, and possibly increasing burden on health services as observed elsewhere [3-5,18]. Using survey data, this paper explores the prevalence and factors associated with self-reported symptoms of injection site infections (abscess or open wound), which are likely to be due to bacterial infection, among IDUs in England, and exploratory cost estimates of their treatment.

## Methods

Data on subject-reported symptoms of an injection site bacterial infection, from a community-recruited survey of IDUs undertaken in England was used [19]. Briefly, an unlinked anonymous community-recruited survey was undertaken between autumn 2003 and summer 2005. This involved recruiting drug users who had injected during the proceeding 28 days using established methods at seven broadly representative locations across England. Participants were recruited either directly from a range of low threshold services, including drop-in centres and needle exchanges, or using indigenous fieldworkers to recruit from the community settings[20]. Those who agreed to take part, by providing verbal consent, were taken through a detailed questionnaire on their demographic, behavioural and drug use characteristics by trained interviewers. They also provided dried blood spot samples for testing for anti-bodies to hepatitis C (anti-HCV), hepatitis B core antigen (anti-HBc), and HIV (anti-HIV), and were offered a £10 acknowledgement. The questionnaire collected information on self-reported symptoms of injecting site infections, and use of health services in response to these. The survey, part of the unlinked anonymous programme, had multi-site approval from the London Research Ethics Committee and from the relevant local committees.

### Analyses

The symptoms of injection site infections considered were reporting either an *'abscess (pus filled swelling)*' or '*open wound/sore*' at an injection site, as these symptoms are most likely to be due to a bacterial infection. All analyses were undertaken in SPSS 14. Variables, such as demographic, drug use and behavioural characteristics, that were found to be univariately associated with these symptoms using χ^2^, were then entered using the forward stepwise procedure in SPSS in to a logistic regression model with inclusion assessed using the likelihood ratio (with the stepwise probability for inclusion of 0.05 and exclusion of 0.1).

### Cost estimation

The cost of treatment was estimated for those IDUs reporting having had an injection site infection in the last year who also reported seeking medical advice. However, specific costs were not collected, and so the cost estimation used standard UK costs from the National Health Service (NHS) reference cost database for 2005–2006 [21] and the 2006 review of 'Unit costs of Health and Social Care' [22]. Also, only limited survey data were collected on healthcare usage. Thus cost estimates were produced that used lower and upper bound estimates for each unit cost and assumed each IDU only had one injection site infection per year.

For those IDUs reporting that they attended an emergency department, the lower and upper quartile costs of an 'Accident and Emergency lower cost investigation' (£67 or £86) was applied [22]. For those who were then admitted to hospital the survey did not collect data on the average duration of hospital stay. Because of this, and because there is little suitable published data on their likely length of stay, two methods were used to produce estimates for the costs of hospital care. The first method simply assigned a low and high cost to each IDU's hospital stay from the range in average unit costs for non-elective hospital based acute care of skin infections (£944–1,556 per patient) [21]. Alternatively, cost projections were produced using the average daily NHS cost for hospital based adult acute care for skin infections (£247–370 per day) [21], and assuming a low and high estimate for the average number of days they stay in hospital (2 or 4 days) [4,8,23]. However, because the cost per day is higher for shorter hospital stays in the NHS cost database, the higher daily cost was applied to the shorter length of stay and vice versa for the longer length of stay. The estimates for an IDU's length of stay were obtained from one Scottish study and two US studies that looked at hospital admissions of IDUs with soft tissue infections [4] or skin infections, abscesses and other health problems [8,23].

For those that sought medical advice but did not attend an emergency department, it was assumed that they consulted their general practitioner (GP). A low and high cost was applied to this by using the upper and lower bound cost for a GP surgery consultation (£18–25 per consultation) from Unit Costs of Health and Social Care [22].

The cost estimates for each care option were combined to produce different cost estimates for the treatment of the IDUs in the study. By multiplying the reported prevalence of injection site infections in the last year with estimates for the number of IDUs in England, 140,000 (only injectors of opiates or crack-cocaine, low estimate) [24] or 215,000 (all IDUs, high estimate) [25], these treatment costs were then scaled up to produce a range of estimates for the yearly cost of treating IDUs with injection site infections.

## Results

This survey recruited 1,058 injectors from seven locations in England: 77% (810/1,054) were male, 18% (193/1,058) aged under 25 years (median age 30 years, range 16 to 72 years), 21% (221/1,056) had been injecting for less than five years (median number of years injecting 9, range < 1 to 41 years), and the prevalence of anti-HCV was 53% (566/1,058), anti-HBc was 32% (332/1,049), and anti-HIV was 0.8% (8/1,058). Almost all had injected an opiate in the previous four weeks (999/1,058), whilst 40% (424/1,058) had injected crack-cocaine and 13% (136/1,058) an amphetamine. The majority (86%, 904/1,056) had ever been homeless (having lived in a hostel, being of no fixed abode, or living on the streets) and over half (59%, 622/1,056) had last been homeless during the proceeding 12 months.

Of the 1,058 participants, 385 (36%) reported having had a symptom of an injection site infection (either an abscess or open wound) in the previous year.

### Factors associated with symptoms injection site infections

Reporting injection site infection in the previous year was associated univariately with a range of factors (Table 1). In the multivariate analysis (Table 1), a number of factors remained associated with reporting an injection site infection: female gender; being aged 25 or over; having injected into legs, groin, and hands in last year; injecting on 14 or more days during the last 4 weeks, cleaning needles/syringes for reuse; injecting crack-cocaine; being anti-HCV positive; and having previously received prescribed substitute drug (i.e. not currently on prescribed opioid substitute drug).

**Table 1:** Factors associated with an injection site infection in last year among injecting drug users: England 2003/05.

		**Yes**	**N ** **(Total = 990)**		**Univariate Odds ** **Ratio with 95% ** **confidence interval**		**Mulitvariate Adjusted ** **Odds Ratio with 95% ** **confidence interval**	
**Gender**	Male	263	755	35%	1.0		1.0	
	Female	102	235	43%	1.4	1.1 – 1.9	1.7	1.2 – 2.4
**Received prescribed substitute drug**	Currently	178	511	35%	1.0		1.0	
	Previously	150	322	47%	1.6	1.2 – 2.2	1.7	1.3 – 2.4
	Never	37	157	24%	0.6	0.4 – 0.9	0.9	0.5 – 1.3
**Age in Years**	<= 24	47	186	25%	1.0		1.0	
	25 – 29	103	273	38%	1.8	1.2 – 2.7	1.6	1.0 – 2.6
	30 – 34	107	258	41%	2.1	1.4 – 3.2	2.0	1.3 – 3.2
	35+	108	273	40%	1.9	1.3 – 2.9	1.9	1.2 – 3.0
**Inject into leg last 4 weeks**	No	212	701	30%	1.0		1.0	
	Yes	153	289	53%	2.6	2.0 – 3.4	2.2	1.6 – 3.1
**Inject into groin last 4 weeks**	No	176	543	32%	1.0		1.0	
	Yes	189	447	42%	1.5	1.2 – 2.0	1.4	1.1 – 1.9
**Inject into hand last 4 weeks**	No	245	759	32%	1.0		1.0	
	Yes	120	231	52%	2.3	1.7 – 3.1	1.9	1.3 – 2.6
**Clean Needle/Syringe for reuse**	No	77	259	30%	1.0		1.0	
	Yes	288	731	39%	1.5	1.1 – 2.1	1.5	1.1 – 2.1
**Inject crack last 4 weeks**	No	188	588	32%	1.0		1.0	
	Yes	177	402	44%	1.7	1.3 – 2.2	1.5	1.1 – 2.0
**Days injected last 4 weeks**	<= 13 days	46	181	25%	1.0		1.0	
	14 – 27 days	84	208	40%	2.0	1.3 – 3.1	1.8	1.2 – 2.9
	28 days	235	601	39%	1.9	1.3 – 2.7	1.5	1.0 – 2.3
**Anti-HCV Positive**	No	135	454	30%	1.0		1.0	
	Yes	230	536	43%	1.8	1.4 – 2.3	1.5	1.1 – 2.0
**Homeless**	Never	36	140	26%	1.0		†	
	Over a year ago	98	266	37%	1.7	1.1 – 2.7		
	In last year	231	584	40%	1.9	1.2 – 2.9		
**Number of years injecting**	<= 4	53	210	25%	1.0		†	
	5 – 9	120	300	40%	2.0	1.3 – 2.9		
	10 – 14	87	227	38%	1.8	1.2 – 2.8		
	15 +	105	253	42%	2.1	1.4 – 3.1		
**Inject into neck last 4 weeks**	No	288	841	34%	1.0		†	
	Yes	77	149	52%	2.1	1.4 – 2.9		
**Prepare drug using citric acid**	No	12	59	20%	1.0		†	
	Yes	353	931	38%	2.4	1.3 – 4.6		
**Times injected last full day**	Once	53	168	32%	1.0		†	
	Twice	77	266	29%	0.9	0.6 – 1.3		
	Thee times	81	203	40%	1.4	0.9 – 2.2		
	4+ times	154	353	44%	1.7	1.1 – 2.5		
**Clean injecting site last 4 weeks**	Never	185	444	42%	1.0		†	
	Sometimes	83	224	37%	0.8	0.6 – 1.1		
	Always	97	322	30%	0.6	0.4 – 0.8		
**Times injected with last needle**	Once	186	549	34%	1.0		†	
	Twice	68	189	36%	1.1	0.8 – 1.5		
	Three times	39	100	39%	1.2	0.8 – 1.9		
	4+ times	72	152	47%	1.8	1.2 – 2.5		
**Ever had voluntary confidential test for hepatitis C**	Yes	257	652	39%	1.4	1.0 – 1.8	†	
	No	108	338	32%	1.0			
**Anti-HBc positive**	No	239	680	35%	1.0		†	
	Yes	126	310	41%	1.3	1.0 – 1.7		

† Variable not in final model.

Note: Reporting an injection site infection in previous year was not associated with: having been imprisoned; having had an overdose; injecting amphetamines; washing hands before injection; injecting into arms; using vitamin c/ascorbic acid, lemon juice or vinegar to dissolve drugs; ever having had voluntary-confidential test for HIV; and uptake of hepatitis B vaccine.

### Factors associated with seeking health care for an injection site infection

Two thirds of those reporting an injection site infection reported seeking health care (68%, 260/382). Seeking health care among those having had injection site infection was associated univariately with the factors shown in Table 2. In multivariate analyses (table 2), seeking health care remained associated with two markers of healthcare utilisation (currently being prescribed substitute drug, and ever having had voluntary-confidential test for hepatitis C); a risk factor for infection (injecting into the groin); and a marker of good hygiene practice (always swabbing injection sites).

**Table 2:** Factors associated with healthcare seeking among injecting drug users reporting an injection site infection in past year: England 2003/05.

		**Yes**	**N ** **(Total = 365)**		**Univariate Odds ** **Ratio with 95% ** **confidence interval**		**Mulitvariate Adjusted ** **Odds Ratio with 95% ** **confidence interval**	
**Received prescribed substitute drug**	Currently	138	182	76%	1.0		1.0	
	Previously	94	147	64%	0.6	0.4 – 0.9	0.5	0.3 – 0.9
	Never	15	36	42%	0.2	0.1 – 0.5	0.3	0.1 – 0.7
**Inject into groin last 4 weeks**	No	100	174	57%	1.0		1.0	
	Yes	147	191	77%	2.5	1.6 – 3.9	2.1	1.3 – 3.4
**Clean injecting site last 4 weeks**	Never	113	184	61%	1.0		1.0	
	Sometimes	58	85	68%	1.3	0.8 – 2.3	1.4	0.8 – 2.6
	Always	76	96	79%	2.4	1.3 – 4.2	2.5	1.4 – 4.6
**Ever had voluntary confidential test for hepatitis C**	Yes	195	257	76%	3.4	2.1 – 5.4	3.5	2.1 – 5.8
	No	52	108	48%	1.0		1.0	
**Number of years injecting**	<= 4	25	53	47%	1.0		†	
	5 – 9	80	120	67%	2.2	1.2 – 4.3		
	10 – 14	64	84	76%	3.6	1.7 – 7.5		
	15 +	78	108	72%	2.9	1.5 – 5.8		
**Inject into arm last 4 weeks**	No	72	94	77%	1.0		†	
	Yes	175	271	65%	0.6	0.3 – 1.0		
**Ever had voluntary confidential test for HIV**	No	71	132	54%	1.0		†	
	Yes	176	233	76%	2.7	1.7 – 4.2		
**Anti-HCV Positive**	No	80	134	60%	1.0		†	
	Yes	167	231	72%	1.8	1.1 – 2.8		

† Variable not in final model.

Note: Seeking health care an injection site infection was found not to be associated with: gender; age; homelessness; having been imprisoned; having had an overdose; cleaning needles and syringes before reuse; injecting crack-cocaine; injecting amphetamines; number of days injecting per month; number times inject per day; using citric acid, vitamin c/ascorbic acid, lemon juice, or vinegar to dissolve drugs; injecting into legs, neck or hands; number times used last needle; washing hands before injection; and uptake of hepatitis B vaccine.

### Heath services use and costs

Almost half of those seeking advice for an injection site infection reported attending an emergency department (47%, 180/381), with over three-quarters of those attending an emergency department reporting admission to hospital (78%, 140/180). Estimates of the numbers of IDUs accessing health services in England in relation to a concern about an injection site infection are given in table 3. These estimates indicate that over 30,000 IDUs are likely to seek health care for injection site infections each year, with at least 18,500 of them being admitted to hospital. Assuming only a single episode per IDU each year, and using conservative unit costs, the annual healthcare costs associated with injection site infections among IDUs in England are estimated to be at least £19.2 million for the lower bound cost per hospital stay, and at least £30.5 million for the upper bound cost per hospital stay (see table 4 for these estimates, both assuming an IDU population of 140,000). This compares with a lower bound estimated cost of at least £15.5 million when daily costs are used and IDUs are assumed to stay in hospital for on average 2 days, and an upper bound cost of at least £20.6 million when IDUs are assumed to stay on average 4 days (both assuming an IDU population of 140,000). Irrespective of the method used, the vast majority of these costs are due to the periods of hospital admission, which makes up over 88% of the total cost estimate.

**Table 3:** Estimated annual numbers of injecting drug users in England seeking healthcare for injection site infections.

	**Proportion of survey respondents**	Estimated number of IDUs in England: 140,000 *	Estimated number of IDUs in England: 215,000 **
**IDUs reporting abscess/open wound in last year**	36%	50,400	77,400
**Those IDUs reporting abscess/open wound in last year seeking care:**	24%	34,272	52,632
**Of these, those that sought care:**			
**other than at an Emergency Department**	8%	10,584	16,254
**at an Emergency Department, not admitted ** **to hospital**	4%	5,211	8,003
**at an Emergency Department, & admitted ** **to hospital**	13%	18,477	28,375

* Estimated number of injectors of heroin or crack-cocaine only, form a study aiming to estimate the total number of heroin and crack-cocaine users [24].

** Estimated number of injecting drug users (all drugs) obtained from a study using an evidence synthesis method to estimate number of injecting drug users [25].

**Table 4:** Estimated annual costs for injecting drug users (IDUs) in England seeking healthcare for injection site infections.

	**Estimated costs in 1,000 pounds sterling (£)**			
	**Low unit costs**†		**High unit costs**†	
Estimated number of IDUs in England	140,000*	215,000**	140,000*	215,000**

Estimated cost of GP consultations	191	293	265	406
Estimated cost of A&E visits	1,587	2,437	2,037	3,129
Estimated cost of hospital bed days	17,442	26,786	28,750	44,151

**Total**	**19,220**	**29,516**	**30,527**	**46,881**

* Low estimate of number of IDUs is the number of heroin or crack-cocaine injectors, form a study aiming to estimate the total number of heroin and crack-cocaine users [24].

** High estimate of number of IDUs is the estimated number of IDUs (all drugs) obtained from a study using an evidence synthesis method to estimate number of injecting drug users [25].

† Low and High Unit costs based on Unit Costs of Health and Social Care 2006 [22] and NHS cost database [21]. Unit cost GP consultation: Low £18 & High £25 [22]. Average Unit cost for a lower cost investigation at Accident and Emergency (Emergency Department): Low £67, & High £86 [22,21]. Average Unit costs per hospital stay were based on the low and high average cost for non-elective adult acute care in a hospital for a skin infection from NHS cost database [21], Alternative estimates are produced using daily unit costs and assuming they stay in hospital for 2 or 4 days [4,8,23]

## Discussion

Symptoms of injection site infections appear to be common amongst IDUs in England. These infections are associated with reuse of syringes, particular injection sites, crack injecting, having hepatitis C infection and being female; with one in seven of the IDUs reporting hospital admission annually. These infections are preventable, yet based on conservative exploratory estimates they may cost the health service in England at least £15.5–19.2 million per annum.

The high prevalence of self-reported injection site infections found in this study, as found elsewhere [4,5], are of concern and highlight the need for interventions which facilitate good injection related hygiene and practice. Firstly, this study found that the injection sites used and the reuse of injecting equipment were associated with injection site infections. Previous studies have found similar associations [9,10]; and also associations with inadequate washing of hands or cleaning of the injection site [5,9,10]; multiple injection attempts before locating a vein, and the use of multiple injection sites [9,10]. The association with femoral ('groin') injection is of particular concern as this has become more common in recent years [26], with indications of increased hospital admissions related to femoral injection among IDUs [13].

Participants injecting crack-cocaine also reported higher levels of injection site infections, as has been observed elsewhere with the injection of cocaine [6,8,36], and heroin and cocaine combinations [10,27]. There is also evidence to suggest crack-cocaine use, which is associated with risky behaviours [26,28], has become more common in the UK [29]. As in other studies, women were more likely to report an injection site infection [6,10,27]. This may reflect a higher awareness of infections, and/or a greater vulnerability to injection site infections among female injectors[30]. The positive association with previously receiving a prescribed substitute drug suggests that those factors leading to people not successfully completing treatment for their drug use may also put them at elevated risk of infection, though this needs further examination.

In this study homelessness – a possible marker for injection in public places – was not associated with injection site infections in the final analyses. This may reflect the relatively broad definition used. In previous studies public or semi-public injecting environments have been related to poor injection hygiene and infections [9,10,31]. In the UK there is increasing concern about public injection and its impact on injecting hygiene [26,28]. Preventive interventions should thus focus on the reuse of injecting equipment, and target those injecting into groins, legs or hand, using crack-cocaine, and female injectors; they may also need to consider the role of the injecting environment [32].

The conservative cost estimate from this study – at least £15.5–19.5 million per annum – indicates that injecting site infections could place a considerable cost burden on the health care system in England. Previous work looking at the costs of problematic drug use, both injecting and non-injecting, in England indicated considerable health care costs of around £500 million per annum [33]. Whilst £25 million of this was estimated to be due to blood borne viruses (HIV, hepatitis B and C) among IDUs, the authors did not separate the costs related to injecting users. They were also not able to separate out the costs related to injecting site infections, and these may not have been fully accounted for in their estimate.

Most of the participants reporting an injection site infection sought medical advice and one third of those with an infection reported being admitted to hospital, many via an emergency department. This is consistent with other studies which suggest that IDUs tend not to seek timely medical care for their injecting-related health problems, often resulting in emergency treatment at considerable cost [8,18,34,35]. The failure to seek earlier treatment probably reflects competing priorities, such as, obtaining money, purchasing and using drugs, barriers to accessing care, and poor compliance with oral antibiotic regimes and follow-up care [8,18,35,36]. Emergency room use (8% of total cost) and hospital admission (> 90% of total cost) account for much of the estimated costs. These costs could be reduced substantially, past studies have shown that earlier health care seeking and targeted prevention can reduce emergency department visits, hospital bed days, and surgical procedures by more than a third [37,38]. Whilst further work needs to identify, develop, and evaluate suitable interventions, our findings suggest a need to focus on improving injection hygiene, the better management of the body sites used for injection, and that IDUs are possibly more likely to seek care if in contact with other services.

It is important to consider the limitations and generalisability of these findings. Self-reported symptoms of injecting site infections were used in this study; however, studies have shown good concordance between self-reported symptoms and clinical diagnosis [18]. The comparative rarity, marginalisation and illegal nature of injecting drug use impedes the recruitment of a representative sample of injectors. This study aimed to minimise sampling biases and maximise representativeness by using an established community sampling strategy [20]. However, in particular, bias might arise from high-risk individuals being more likely to be captured in the survey as a result of them making greater use of the needle exchanges, and other settings, where sampling occurred. As a consequence the cost estimates could be over estimates. The cost estimates were however calculated conservatively. Firstly, they assume that IDUs seeking care only did so once each year – yet some will have more than one such episode of care per annum and a Canadian study found that half of IDUs admitted to hospital for soft-tissue and systemic infections had multiple admissions [8]. If this was the case then the estimated costs of injection site infections in England could be more than 50% greater, or at least £23 million per annum. Four days for a long hospital stay may also be conservative, as those with a serious systemic infection could stay much longer, as has found in hospitals in the USA (3 to 13 days) [4,34,39]. However, the available data on length of IDU hospital stays is limited, with the only UK data being 20 years old and no clinical data were collected as part of this study. The cost estimates also excluded any periods of care in high dependency units, or other high cost services, which may be required for treatment of the more severe infections. Even so, the costing, and other findings, presented here are tentative, and a more rigorous economic evaluation is required that includes UK specific data on the average hospital stay for IDUs with infection site infections and the costs of these.

## Conclusion

Taken together these findings suggest injection site infections are common experiences among IDUs in England, and that the resultant health care costs are likely to be substantial. However, the size of these costs needs to be more fully determined. Further research is also needed to explore issues around the validity of self-reports and the relationship between these reported symptoms of infection, the severity of the infection, and health seeking behaviour.

## Abbreviations

anti-HBc: antibodies to hepatitis B core antigen; anti-HCV: antibodies to hepatitis C; anti-HIV: antibodies to HIV; IDUs: Injecting Drug Users, NHS: National Health services; GP: General Practitioner; UK: United Kingdom; USA: United States of America.

## Competing interests

The authors declare that they have no competing interests.

## Authors' contributions

All authors contributed to preparing the manuscript, with VH coordinating and JK assisting. VH, MH & FN contributed to the design of the study, with VH leading the study implementation. Analyses were undertaken by VH and PV, supported by JK.

## Pre-publication history

The pre-publication history for this paper can be accessed here:



## References

[B1] Cherubin C, Sapira J (1993). The medical complications of drug addiction and the medical assessment of the intravenous drug user. Ann Intern Med.

[B2] Del Guidice P (2004). Cutaneous complication of intravenous drug abuse. Br J Dermatol.

[B3] Darke S, Ross J, Kaye S (2001). Physical injecting sites among injecting drug users in Sydney, Australia. Drug Alcohol Depend.

[B4] Takahasi T, Merrill J, Boyko E, Bradley K (2003). Type and location of injection drug use-related soft tissue infections predict hospitalization. J Urban Health.

[B5] Biswanger I, Kral A, Bluthenthal R, Rybold D, Edlin B (2000). High prevalence of abscesses and cellulitis among community-recruited injection drug users in San Fransisco. Clin Infect Dis.

[B6] Lloyd-Smith E, Kerr T, Hogg RS, Li K, Montaner JS, Wood E (2005). Prevalence and correlates of abscesses among a cohort of injection drug users. Harm Reduct J.

[B7] Kerr T, Wood E, Grafstein E, Ishida T, Shannon K, Lai C, Montaner J, Tyndall MW (2005). High rates of primary care and emergency department use among injection drug users in Vancouver. J Public Health (Oxf).

[B8] Palepu A, Tyndall M, Leon H, Muller J, O'Shaughnessy M, Schechter M, Anis A (2001). Hospital utilization and costs in a cohort of injection drug users. CMAJ.

[B9] Vlahov D, Sullivan M, Astemborski J, Nelson KE (1992). Bacterial infections and skin cleaning prior to infection among intreavenous drug users. Public Health Rep.

[B10] Murphy E, DeVita D, Lui H, Vittinghoff E, Leung P, Ciccarone D, Edlin B (2001). Risk factors for skin and soft-tissue abscesses among Injection Drug Users: A case-control study. Clin Infect Dis.

[B11] Health Protection Agency, Health Protection Scotland, National Public Health Service for Wales, CDSC Northern Ireland, CRDHB, and the UASSG (2006). Shooting Up: Infections among injecting drug users in the United Kingdom 2005.

[B12] Lamagni TL, Neal S, Keshishian C, Alhaddad N, George R, Duckworth G, Vuopio-Varkila J, Efstratiou A (2008). Severe *Streptococcus pyogenes *infections, United Kingdom, 2003–2004. Emerg Infect Dis.

[B13] Irish C, Maxwell R, Dancox M, Brown P, Trotter C, Verne J, Shaw M (2007). Skin and soft tissue infections and vascular disease among drug users, England [letter]. Emerg Infect Dis.

[B14] Health Protection Agency (2003). Methicillin resistant Staphylococcus aureus (MRSA) in injecting drug users. CDR Weekly.

[B15] Kearns AM, Rathmann IR, Holmes A, Pitt TL, Cookson BD (2004). An unusual clone of MRSA causing infection in injecting drug users. J Infect.

[B16] Beeching NJ, Crowcroft NS (2005). Tetanus in injecting drug users. The latest Clostridium infection to threaten injectors in Britain. BMJ.

[B17] Akbulut D, Dennis J, Gent M, Grant KA, Hope V, Ohai C, McLauchlin J, Mithani V, Mpamugo O, Ncube F (2005). Outbreak report: Wound botulism in injectors of illicit drugs: upsurge in cases in England during 2004. EuroSurveillance Monthly.

[B18] Morrison A, Elliott L, Gruer L (1997). Injecting-related harm and treatment seeking behaviour among injecting drug users. Addiction.

[B19] Hickman M, Hope V, Brady T, Madden P, Jones S, Honour S, Holloway G, Ncube F, Parry J (2007). Hepatitis C (HCV) prevalence, and injecting risk behaviour in multiple sites in England in 2004. J Viral Hepat.

[B20] Griffiths P, Gossop M, Powis B, Strang J (1993). Reaching hidden populations of drug users by privileged access interviewers: methodological and practical issues. Addiction.

[B21] National Health Service (2006). NHS reference costs 2005–06.

[B22] Curtis L, Netten A (2006). Unit costs of Health and Social Care.

[B23] Horn E, Henderson H, Forrest J (1987). Admissions of drug addicts to a general hospital: A retrospective study in the northern district of Glasgow. Scott Med J.

[B24] Hay G, Gannon M, MacDougall J, Millar T, Eastwood C, McKeganey N, Singleton N, Murray R, Tinsley L Local and national estimates of the prevalence of opiate use and/or crack cocaine use (2004/05). Measuring different aspects of problem drug use: methodological developments London, Home Office, 2006 Home Office Online Report, 16/06 ISBN-13: 978 1 84726 123 6.

[B25] De Angelis D, Sweeting M, Ades AE, Hickman M, Hope V, Ramsay M An evidence synthesis approach to estimating Hepatitis C Prevalence in England and Wales. Stat Methods Med Res.

[B26] Rhodes T, Stoneman A, Hope V, Hunt N, Martin a, Judd A (2006). Groin injecting in the context of crack cocaine and homelessness: From 'risk boundary' to 'acceptable risk'?. Int J Drug Policy.

[B27] Spijkerman I, van Ameijden EJ, Mientjes G (1996). Human immunodeficiency virus and other risk factors for skin abscesses and endocarditis among injection drug users. J Clin Epidemiol.

[B28] Rhodes T, Briggs D, Kimber J, Jones S, Holloway G (2007). Crack-heroin speedball injection and its implications for vein care: qualitative study. Addiction.

[B29] Hope VD, Hickman M, Tilling K (2005). Capturing crack-cocaine use: Estimating the prevalence of Crack-cocaine use in London using capture-recapture with covariates. Addiction.

[B30] Topp L, Iversen J, Conroy A, Salmon A, Maher L (2008). Prevalence and predictors of injecting-related injury and disease among clients of Australia's needle and syringe programs. Aust N Z Journal of Public Health.

[B31] Broadhead RS, Kerr TH, Grund J, Altice FL (2002). Safer injection facilities in North America: Their place in public policy and health initiatives. J Drug Issues.

[B32] Rhodes T, Kimber J, Small W, Fitzgerald J, Kerr T, Hickman M, Holloway G (2006). Public injecting and the need for 'safer environment interventions' in the reduction of drug-related harm. Addiction.

[B33] Gordon L, Tinsley L, Godfrey C, Parrott S, Singleton N, Murray R, Tinsley L The economic and social costs of Class A drug use in England and Wales, 2003/04. Measuring different aspects of problem drug use: methodological developments London, Home Office, 2006 Home Office Online Report, 16/06 ISBN-13: 978 1 84726 123 6.

[B34] Ciccarone D, Bamberger J, Kral A, Edlin B, Hobart C, Moon A, Murphy E, Bourgois P, Harris H, Young D (2001). Soft tissue infections among injection drug users-San Francisco, California, 1996–2000. MMWR CDC Surveill Summ.

[B35] French M, McGeary K, Chitwood DD, McCoy CB (2000). Chronic illicit drug use, health services utilisation and the cost of medical care. Soc Sci Med.

[B36] van Beek I, Dwyer R, Malcom A (2001). Cocaine injecting: the sharp end of drug related harm!. Drug Alcohol Rev.

[B37] Harris H, Young D, Organ C (2002). Care of injection drug users with soft tissue infections in San Fransisco. Arch Surg.

[B38] Grau L, Arevalo S, Catchpool C, Heimer R (2002). Expanding harm reduction services through a wound and abscess clinic. Am J Public Health.

[B39] Schnall SB, Holtom PD, Lilley JC (1994). Abscesses secondary to parenteral abuse of drugs. A study of demographic and bacteriological characteristics. J Bone Joint Surg Am.

